# Dimensional Priming Reprograms Adipose-Derived Stromal Cells to Promote Pancreatic Cancer Progression

**DOI:** 10.3390/cancers18030460

**Published:** 2026-01-30

**Authors:** Bo Han, Zhi Yang, Shuqing Zhao, Thomas Schmittgen, Jamel Ali, Ba Xuan Hoang

**Affiliations:** 1 Departments of Surgery, University of Southern California, Los Angeles, CA 90033, USA; 2 Biomedical Engineering, University of Southern California, Los Angeles, CA 90033, USA; 3 College of Pharmacy, University of Florida, Gainesville, FL 32610, USA; 4 Department of Chemical and Biomedical Engineering, FAMU-FSU College of Engineering, Tallahassee, FL 32310, USA

**Keywords:** adipose-derived stromal cells, dimensional priming, stromal plasticity, 3D culture systems, matrix mechanics, PDAC

## Abstract

Background: The tumor microenvironment (TME) plays a central role in pancreatic ductal adenocarcinoma (PDAC) progression, yet how mechanical cues shape stromal cell behavior remains poorly defined. Here, we investigate how dimensional priming of adipose-derived stromal cells (ADSCs) alters their immunomodulatory functions and subsequent impact on PDAC growth. Methods: ADSCs were cultured under two-dimensional (2D) or three-dimensional (3D) conditions and evaluated using in vitro co-culture systems with PDAC organoids and in vivo xenograft models. Stromal phenotype, cytokine secretion, tumor growth, invasion, and immune cell infiltration were assessed. Results: ADSCs cultured in three-dimensional (3D) hydrogels exhibited reduced Caveolin-1 (CAV-1) expression and reprogramming toward a stress-adapted, CAF-like phenotype compared with two-dimensional (2D) cultures. In vitro, 2D-primed ADSCs constrained PDAC organoid growth, increased MMP-2 activity, and required direct cell–cell contact to suppress tumor viability. By contrast, 3D-primed ADSCs preserved organoid structure but markedly enhanced tumor cell migration through soluble factors, accompanied by increased IL-6 and TNF-α and reduced IL-10 secretion during co-culture. In vivo, 3D-primed ADSCs promoted the largest tumors with aggressive invasion and loss of Col-Tgel containment associated with tumor expansion, whereas 2D-primed ADSCs suppressed tumor growth and maintained gel boundaries. Immunohistochemistry confirmed elevated Ki-67 in tumors containing 3D-primed ADSCs, while macrophage infiltration (F4/80^+^) was highest in 2D-primed tumors and lowest in 3D-primed tumors. Conclusions: Dimensional priming fundamentally reprograms ADSC phenotype and alters their stromal–immune interactions, generating a tumor-permissive state that accelerates PDAC progression. These findings identify mechanical cues as critical regulators of stromal plasticity and highlight dimensional priming as a potentially targetable axis within the PDAC microenvironment.

## Simple Summary

Pancreatic cancer is difficult to treat in part because tumor cells interact closely with surrounding supportive cells and tissue, known as the tumor microenvironment. These supportive cells can either restrain or promote cancer growth depending on their physical and biological state. In this study, we examined how growing adipose-derived stromal cells under flat (two-dimensional) or three-dimensional conditions changes their behavior and their effects on pancreatic cancer. We found that stromal cells primed in three-dimensional environments promote tumor growth, invasion, and tissue breakdown in animal models, whereas cells primed under flat conditions tend to suppress tumor expansion. These findings highlight how physical cues in the tumor environment can reprogram supportive cells and strongly influence cancer progression. Understanding these interactions may help guide future strategies to target the tumor microenvironment in pancreatic cancer.

## 1. Introduction

Pancreatic ductal adenocarcinoma (PDAC) is among the most lethal solid tumors, with a five-year survival rate of less than 12% despite advances in surgery and systemic therapy [1]. Its aggressive biology is closely linked to the tumor microenvironment, which is characterized by a dense desmoplastic stroma comprising fibroblasts, mesenchymal stromal cells, immune cells, and extracellular matrix (ECM) components [2,3]. Far from being a passive scaffold, the stroma plays an active role in tumor progression, invasion, and therapeutic resistance by providing structural support, biochemical cues, and immunomodulatory signals [4].

Cancer-associated fibroblasts (CAFs) are a major stromal population in PDAC and exhibit marked heterogeneity, with subsets exerting tumor-restraining as well as tumor-promoting functions [4]. Similarly, multipotent stromal cells derived from bone marrow or adipose tissue have been shown to modulate tumor growth through paracrine signaling, ECM remodeling, and regulation of immune cell recruitment [5,6]. Adipose-derived stromal cells (ADSCs) are abundant, easily accessible, and display strong immunomodulatory capacity, but their contribution to PDAC biology remains controversial. Prior studies report tumor-suppressive effects of ADSCs, including direct induction of pancreatic cancer cell death [7], as well as tumor-supportive or context-dependent effects mediated by immunomodulation and metabolic crosstalk [8,9].

The dimensionality and mechanical properties of the stromal niche represent key contextual cues that may explain these discrepancies. Conventional two-dimensional (2D) culture systems do not recapitulate the physical architecture or stiffness of in vivo tissues. In contrast, three-dimensional (3D) hydrogels better mimic the tumor milieu and can profoundly reprogram stromal cell phenotype through mechanotransduction pathways [10,11]. For example, stiff matrices have been shown to promote fibroblast activation, stemness, and resistance to apoptosis [12], while soft matrices may restrain tumor-promoting functions [13]. Whether such “dimensional priming” alters ADSC behavior and its impact on PDAC remains poorly defined. Accordingly, we focused on selected cytoskeletal, stromal, and mechanosensitive markers (e.g., F-actin, Desmin, and Caveolin-1) and related genes to capture key changes in ADSC activation, mechanotransduction, and immunomodulatory state induced by dimensional priming.

In this study, we investigated how 2D- versus 3D-primed ADSCs affect PDAC progression using in vitro co-culture systems and in vivo xenograft models. We show that dimensional priming alters ADSC morphology, cytokine secretion, and immunomodulatory properties, which in turn influence tumor growth, invasion, and macrophage recruitment. Our findings highlight dimensional priming as a critical determinant of stromal–tumor interactions and suggest that mechanical cues within the microenvironment may be exploited to modulate stromal support of PDAC.

## 2. Methods

### 2.1. Cell Culture and Dimensional Priming

Human adipose-derived stromal cells (ADSCs; Lonza, Walkersviller, MD, USA) were expanded in DMEM supplemented with 10% FBS and 1% penicillin–streptomycin. For 2D priming, ADSCs were maintained in standard monolayer culture on tissue culture plastic. For 3D priming, ADSCs were embedded in a 10 kPa transglutaminase-crosslinked gelatin hydrogel (Col-Tgel) [14,15] for 7 days unless otherwise noted. Pancreatic cancer CFPAC-1 cells (CRL-1918, ATCC, Manassas, VA, USA) were cultured in RPMI supplemented with 10% FBS and 1% penicillin–streptomycin.

### 2.2. Conditioned Media Preparation and Co-Culture Experiments for Direct Co-Culture

Primed ADSCs and CFPAC-1 cells were embedded together in 3D hydrogel at a 1:1 ratio. For conditioned medium experiments, ADSCs were primed separately in 2D or 3D, washed, and cultured in serum-free medium for 48 h. Conditioned media (CM) were collected, centrifuged to remove debris, and stored at −80 °C until use. For treatment experiments, CM was added to recipient cultures at 30% (*v*/*v*) in complete DMEM. Control groups received an equivalent volume (30% *v*/*v*) of fresh DMEM, ensuring identical medium composition and serum concentration across all conditions.

### 2.3. Organoid Morphology and Migration Assays

Organoid morphology was assessed by phase-contrast microscopy at 7, 14, and 21 days. Migration was quantified from phase-contrast images by defining a 500 µm peri-gel band outside the organoid gel using ImageJ, version 1.51f (2024) Single cells within this region were segmented by thresholding and particle analysis (20–400 µm^2^, circularity 0.2–1.0). Data were expressed as migrated cell coverage per gel perimeter length.

### 2.4. Cell Viability Assays

Cell viability in co-culture and CM experiments was measured using the Cell Counting Kit-8 (CCK-8; Dojindo, Rockville, MD, USA) following manufacturer’s instructions. Absorbance at 450 nm was measured using a microplate reader and normalized to control conditions.

### 2.5. Cell Death Assays

Cell death was assessed by ethidium homodimer-1 (EthD-1) staining (Invitrogen, Walthman, MA, USA) following the manufacturer’s protocol. CFPAC-1 cells were imaged by fluorescence microscopy, and EthD-1–positive (red) versus total cell area (black) was quantified using ImageJ (version 1.54f) to calculate red/black ratios. Senescence-associated β-galactosidase staining was performed using a kit from Cell Signaling Technology (Danvers, MA, USA) with blue-positive cells scored at days 3, 7, and 14.

### 2.6. Reactive Oxygen Species (ROS) Staining

Intracellular ROS was detected using CellROX Green (Invitrogen, Thermo Fisher Scientific, Walthman, MA, USA). ADSCs were cultured in 3D matrices for 3, 7, or 14 days, incubated with 5 µM CellROX Green for 30 min at 37 °C, washed, and imaged under identical exposure conditions.

### 2.7. Immunofluorescence and Immunohistochemistry

For in vitro staining, ADSCs were fixed in 4% paraformaldehyde and stained with antibodies against CAV-1 (Abcam, Cambridge, MA, USA, 1:200), OCT4 (Cell Signaling, Danvers, MA, USA, 1:200), or desmin (Sigma, St. Louis, MO, USA, 1:200). Phalloidin (Invitrogen, 1:200) and DAPI were used for cytoskeletal and nuclear counterstaining. Images were acquired on a Zeiss fluorescence microscope. For selected co-culture experiments, CFPAC-1 tumor cells were pre-labeled with MitoTracker Red and ADSCs with MitoTracker Green prior to embedding, to facilitate cell-type identification during morphological analysis.

### 2.8. Cytokine Profiling

Cytokine secretion was analyzed using a multiplex ELISA panel (Bio-Plex, Bio-Rad, Hercules, CA, USA) for IL-4, IL-6, IL-10, IL-1RA, and TNF-α. Media were collected from ADSC monocultures or ADSC–CFPAC co-cultures after 7 days, centrifuged, and analyzed per manufacturer’s instructions. Cytokine concentrations were normalized to the total number of viable cells at the time of collection. For comparability across conditions, all experiments were initiated with identical total cell numbers (monoculture: 2 × cells; co-culture: 1 × CFPAC-1 cells + 1 × primed ADSCs).

### 2.9. Gene Expression Analysis

RNA was isolated using the RNeasy Mini Kit (Qiagen, Germantown, MD, USA), and cDNA synthesized with iScript (Bio-Rad, Hercules, CA, USA). Quantitative PCR was performed with SYBR Green Master Mix (Applied Biosystems, Waltham, MA, USA) on a CFX96 system (Bio-Rad). Primers targeted MMP1, RUNX2, SOX9, ABCG2, SPP1, COL1A2, HIF1A, and TGF-β1, normalized to GAPDH. Fold changes were calculated using the ΔΔCt method.

### 2.10. Gelatin Zymography

Supernatants from ADSC–CFPAC co-cultures were collected at day 14 and subjected to gelatin zymography to measure pro- and active MMP2. Band intensity was quantified by ImageJ densitometry and normalized to control cultures.

### 2.11. In Vivo Xenograft Studies

All animal procedures were approved by the Institutional Animal Care and Use Committee (IACUC) of the University of Southern California (Protocol No. 21580) and conducted in accordance with institutional and NIH guidelines for the care and use of laboratory animals. Nude mice (6–8 weeks old, *n* = 6 per group) were injected subcutaneously with CFPAC-1 cells alone or mixed with equal numbers of 2D- or 3D-primed ADSCs (1 × 10^6^ cells each). Tumor growth was measured weekly with calipers, and tumor volumes were calculated as (length × width^2^)/2. Mice were sacrificed at 28 days, and tumors excised, weighed, fixed, and processed for histology and immunohistochemistry.

### 2.12. Statistical Analysis

All experiments were performed in triplicate unless otherwise noted. Data are presented as mean ± SEM. Comparisons between groups were performed using one-way ANOVA with Tukey’s post hoc test or two-tailed Student’s *t*-test, as appropriate. *p* < 0.05 was considered statistically significant.

## 3. Results

### 3.1. Human ADSCs Exhibit Matrix-Dependent Phenotypic Plasticity

**Figure 1:**
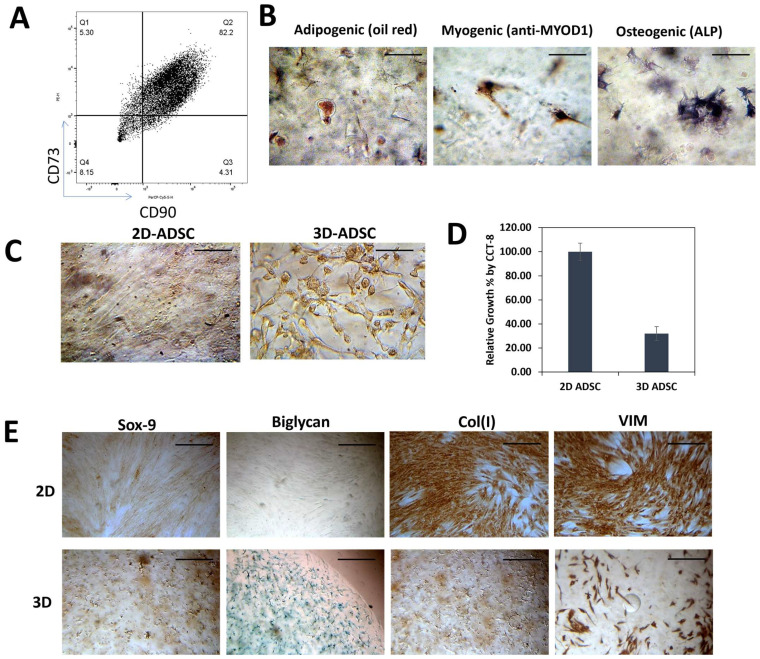
Characterization of ADSCs and evaluation of their phenotypic properties under 2D and 3D culture conditions. (**A**) Flow cytometry analysis confirming stromal identity of commercial human ADSCs, with positive expression of canonical markers CD90 and CD73. (**B**) Induction of multilineage differentiation in 2D-ADSCs. Osteogenesis was assessed by Alizarin Red staining, adipogenesis by Oil Red O staining, and chondrogenesis by Alcian Blue staining. (**C**) Representative images showing ADSC morphology under 2D monolayer versus 3D matrix (~10 kPa) culture. (**D**) Cell proliferation was assessed by CCK-8 assay after 7 days of culture in 2D or 3D conditions. Values are presented as relative growth percentage (mean ± SEM). *n* = 3 independent experiments. (**E**) Immunohistochemical analysis of matrix-related and phenotypic markers. ADSCs were cultured under 2D or 3D conditions, fixed, and stained with antibodies against type I collagen, vimentin, Sox9, and biglycan to evaluate ECM-associated and lineage-related marker expression. Scale bars: 100 μm unless otherwise indicated. Representative images are shown from three independent experiments.

We first validated the stromal identity of commercial human ADSCs by flow cytometry, which confirmed expression of canonical markers CD90 and CD73 (Figure 1A) [16]. Their multipotent potential was validated through directed differentiation, with positive staining for osteogenesis (Alizarin Red), adipogenesis (Oil Red O), and chondrogenesis (Alcian Blue) (Figure 1B) [17]. To assess the impact of dimensionality, ADSCs were cultured either on 2D plastic or embedded in 3D Col-Tgel hydrogels (~10 kPa stiffness). Morphological analysis showed distinct differences: 2D-cultured ADSCs exhibited an elongated, fibroblast-like morphology with extended podia, whereas 3D-cultured cells displayed a rounded shape consistent with cytoskeletal reorganization (Figure 1C) [17]. Dimensionality also influenced cell growth and marker expression. Proliferation was significantly reduced in 3D culture, with an estimated 1.8-fold increase in doubling time relative to 2D (*p* < 0.05, Figure 1D) [18]. Immunohistochemical staining showed that 2D-primed ADSCs expressed higher COL1A1 and vimentin, consistent with an active mesenchymal state. In contrast, 3D priming upregulated Sox9 protein and biglycan, linked to ECM remodeling and stromal plasticity (Figure 1E). Collectively, these findings indicate that matrix dimensionality strongly regulates ADSC morphology, proliferation, and expression of ECM-related and lineage-associated markers [19], highlighting dimensional priming as a mechanism that may reprogram stromal function in the PDAC microenvironment.

### 3.2. Dimensional Priming of ADSCs Differentially Regulates PDAC Organoid Behavior

**Figure 2:**
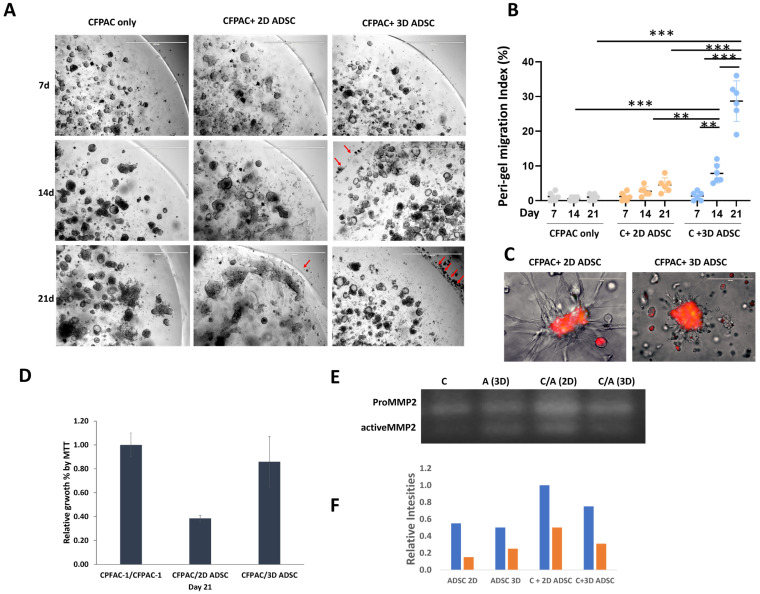
Dimensional priming of ADSCs differentially regulates PDAC organoid behavior. CFPAC-1 cells were co-cultured with either 2D- or 3D-primed ADSCs in a 3D hydrogel matrix system and monitored over 21 days. (**A**) Phase-contrast images showing organoid morphology at days 7, 14, and 21. Dome structure, gel integrity, and cell migration (red arrows) across the gel boundary were evaluated. Higher-magnification inspection of the peri-gel region indicates that migrating elements consist primarily of single cells with occasional small clusters, rather than organized tumor organoids. Scale bar = 1000 µm. (**B**) Peri-gel migration was quantified by defining a standardized 500 µm region surrounding the gel boundary. Cells outside the gel were segmented using ImageJ based on size (20–400 µm^2^) and circularity (0.2–1.0), and migration was expressed as total migrated cell coverage normalized to gel perimeter length. ** *p* < 0.01, *** *p* < 0.001 (**C**) CFPAC-1 cells were pre-labeled with MitoTracker Red and ADSCs with MitoTracker Green prior to co-culture; images are shown as merged channels for morphological context, as the ADSC green signal was comparatively weak. Morphological interactions between ADSCs and tumor cells were assessed, with attention to podia/lamellipodia formation and their effect on cancer cell distribution. (**D**) Cell viability was measured on day 21 using the CCK-8 assay and normalized to CFPAC-1 controls, reflecting total co-culture metabolic activity. *n* = 3 biological replicates. (**E**) Matrix degradation was evaluated by gelatin zymography of culture supernatants. (**F**) Densitometric analysis of zymography was performed to quantify pro- and active MMP2 band intensity, normalized to control.

To assess how dimensional priming of ADSCs alters their influence on pancreatic cancer cells, CFPAC-1 organoids were co-cultured with either 2D- or 3D-primed ADSCs in a 3D matrix system (Figure 2A). Organoids formed efficiently across all conditions by day 7. By day 14, divergent patterns emerged: co-culture with 2D-primed ADSCs was associated with collapse of organoid domes, progressive gel degradation, and visible distortion of the gel boundary. In contrast, 3D-primed ADSCs preserved dome integrity but promoted extensive cancer cell dispersal beyond the gel boundary. We next quantified migration into the peri-gel zone across time points (Figure 2B). At day 7, migration remained minimal and did not differ among groups. By day 14 and 21, however, 3D-primed ADSC co-cultures displayed significantly greater peri-gel migration (red arrows) compared with both control and 2D-primed ADSC conditions (*p* < 0.01). Although a trend toward increased migration was noted in the 2D-primed ADSC co-culture group at later stages, this did not reach statistical significance. Within the 3D group, migration on day 14 and day 21 was significantly elevated relative to day 7 (*p* < 0.01), indicating a progressive, time-dependent effect. Morphological analysis further highlighted contrasting stromal behaviors (Figure 2C). 2D-primed ADSCs extended prominent podia (ADSC-derived signal, green channel in merged images) that spatially interacted with CFPAC-1 tumor cells (red) within the gel, whereas 3D-primed ADSCs exhibited shorter lamellipodia and a more dispersed tumor cell distribution. Based on pre-labeling and cell morphology, the prominent podia observed in the CFPAC + 2D-primed ADSC condition were attributed to ADSCs. Cell viability assays on day 21 confirmed these divergent outcomes (Figure 2D): co-culture with 2D-primed ADSCs reduced CFPAC-1 viability to ~68% of control (*p* < 0.05), while 3D-primed ADSCs preserved or slightly increased viability (~108% of control, n.s.). Mechanistically, zymography demonstrated that MMP2 activity was strongly elevated in 2D-primed ADSC co-cultures, with both pro- and active MMP2 bands increased approximately twofold compared to control (*p* < 0.05; Figure 2E,F). This correlated with the pronounced gel degradation observed in these cultures. By contrast, 3D-primed ADSCs did not enhance MMP2 activity, yet still drove robust cancer cell migration, consistent with a mechanism mediated by soluble pro-migratory factors rather than matrix breakdown. Collectively, these findings indicate that dimensional priming produces opposing influences on PDAC organoid behavior: 2D priming reduces viability but accelerates matrix degradation, whereas 3D priming maintains organoid structure while enhancing cell migration and dispersal [20].

### 3.3. Distinct Requirements for ADSC-Mediated Effects on PDAC Migration and Viability

**Figure 3:**
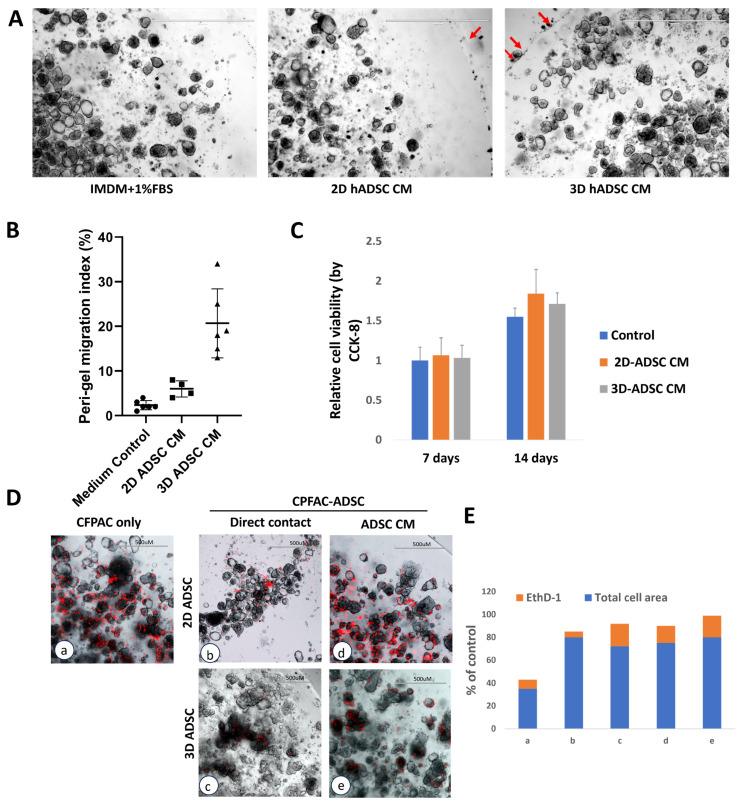
Distinguishing contact-dependent and soluble factor-mediated effects of ADSCs on PDAC migration and viability. CFPAC-1 cells were cultured either in conditioned medium (CM) from 2D- or 3D-primed ADSCs or in direct co-culture with ADSCs. (**A**) Phase-contrast images at day 14 showing organoid morphology and migration (red arrows). Scale bar = 1000 µm. (**B**) Semi-quantitative migration analysis. Data are expressed as migrated cell coverage normalized to gel perimeter length. (**C**) Cell viability was measured after 14 days of CM exposure using the CCK-8 assay, normalized to control medium. (**D**) Cell death was assessed by ethidium homodimer-1 (EthD-1) staining in both CM and co-culture conditions with primed ADSC in 3D culture. (**a**) CFPAC only; (**b**) CFPAC-1 co-cultured with 2D-primed ADSCs; (**c**) CFPAC-1 co-cultured with 3D-primed ADSCs; (**d**) CFPAC-1 treated with 2D-ADSC-primed CM; (**e**) CFPAC-1 treated with 3D-ADSC-primed CM. Total cell area (black) and EthD-1–positive area (red) were quantified, and ratios of positive/total pixels were calculated. Representative images show spontaneous positivity in cancer-only controls, increased staining and reduced cell density in 2D-ADSC direct co-cultures, fewer positive cells in 3D-ADSC direct co-cultures, and no significant changes in CM conditions. (**E**) Semi-quantitative EthD-1 analysis confirming that tumor cell inhibition occurred only in direct contact with 2D-primed ADSCs, whereas 3D-primed ADSCs reduced cell death relative to controls.

To determine whether ADSC effects on PDAC cells are mediated by soluble factors or require direct contact, we compared conditioned medium (CM) with direct co-culture experiments. To exclude potential confounding effects of serum dilution, all CM-treated and control groups were maintained under identical serum conditions by volume-matched supplementation. At day 14, CM from 3D-primed ADSCs induced robust migration of single tumor cells beyond the hydrogel perimeter, whereas control medium and 2D-ADSC CM supported only minimal extragel migration (Figure 3A). Semi-quantitative analysis of a 500 µm peri-gel band confirmed significantly greater migration in the 3D-ADSC CM group compared with both control and 2D-ADSC CM (Figure 3B), indicating that soluble factors secreted by 3D-primed ADSCs are sufficient to promote PDAC dispersal. In contrast, CM from 2D-primed ADSCs did not reproduce the growth-suppressive effect observed in direct co-culture. Cell viability assays at day 14 showed no significant differences among control, 2D-ADSC CM, and 3D-ADSC CM (Figure 3C), suggesting that the inhibitory effect of 2D-primed ADSCs requires direct cell–cell contact or short-range signaling lost in CM. To validate this, cell death was assessed using ethidium homodimer-1 (EthD-1) staining under both CM and co-culture conditions. Cancer-only cultures displayed spontaneous EthD-1 positivity, with necrotic cells accumulating in the gel center and highly proliferative cells at the periphery (Figure 3D(a)). Direct co-culture with 2D-primed ADSCs markedly reduced the total cell number while exhibiting lower EthD-1–positive staining compared with CFPAC-only control (Figure 3D(b)), consistent with contact-dependent growth suppression rather than increased cell death. By contrast, direct co-culture with 3D-primed ADSCs showed far fewer EthD-1–positive cells (Figure 3D(c)), consistent with preserved viability. In CM cultures (Figure 3D(d,e)), the proportion of EthD-1–positive cells remained similar to controls, indicating no significant effect. Semi-quantitative analysis confirmed that tumor cell inhibition occurred only in direct contact with 2D-primed ADSCs, whereas 3D-primed ADSCs reduced cell death relative to controls (Figure 3E). Together, these findings indicate that suppression of PDAC viability by 2D-primed ADSCs depends on direct cellular interactions, while the pro-migratory effect of 3D-primed ADSCs is mediated by soluble factors stable in conditioned medium.

### 3.4. Dimensional Priming Reprograms ADSCs, Induces Time-Dependent Adaptation, and Reshapes Cytokine Responses During Co-Culture with Tumor Cells

**Figure 4:**
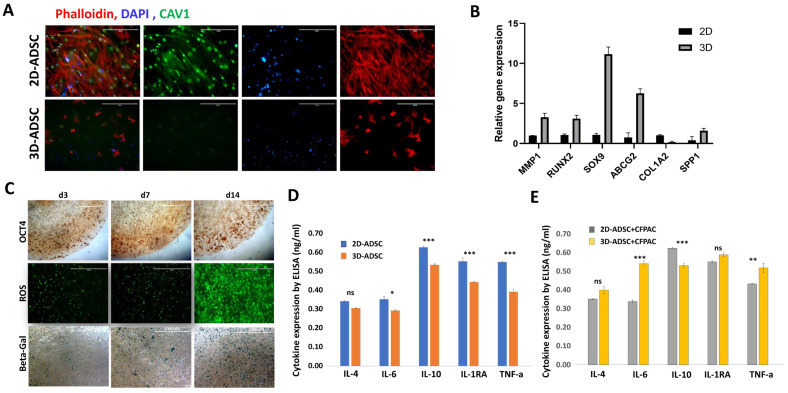
Dimensional priming reprograms ADSCs, induces time-dependent adaptation, and reshapes cytokine responses during co-culture with tumor cells. (**A**) Immunofluorescence staining of ADSCs cultured in 2D or 3D matrices for 7 days, showing phalloidin (red), nuclei (DAPI, blue), and CAV-1 (green). 3D-primed ADSCs exhibited reduced CAV-1 expression compared with 2D cultures. Phalloidin was used to label filamentous actin (F-actin) to visualize cytoskeletal organization. Scale bar = 200 µm. (**B**) Gene expression profiling of ADSCs after 7 days of 2D or 3D culture. qPCR was performed for MMP1, RUNX2, SOX9, ABCG2, SPP1, and COL1A2, normalized to GAPDH. (**C**) Time-course adaptation assays of ADSCs cultured in 3D matrices (~10 kPa). OCT4 immunostaining (stemness), ROS staining (oxidative stress), and senescence-associated β-galactosidase staining were performed at days 3, 7, and 14 to assess time-dependent changes. (**D**) Cytokine secretion in ADSC monocultures. Human IL-4, IL-6, IL-10, IL-1RA, and TNF-α levels were measured by multiplex ELISA after 7 days in 2D or 3D culture, normalized to cell number. (**E**) Cytokine secretion in co-culture with CFPAC-1 cells. Conditioned media were collected at day 7, and cytokines were quantified by multiplex ELISA, comparing 2D- versus 3D-primed ADSCs. * *p* < 0.05, ** *p* < 0.01, *** *p* < 0.001.

To investigate how dimensional priming alters ADSC phenotype, we first examined structural and molecular changes under 2D versus 3D culture. Immunofluorescence staining revealed a marked reduction in CAV-1 expression in 3D-primed ADSCs compared with 2D cultures, consistent with altered membrane signaling and mechanosensing (Figure 4A). Gene expression analysis further demonstrated that 3D priming upregulated several stroma- and stemness-associated markers, including MMP1, RUNX2, SOX9, ABCG2, and SPP1, while COL1A2 was downregulated (Figure 4B), suggesting a shift toward a CAF-like and pro-migratory state. We next performed time-course studies to assess how ADSCs adapt to the 3D microenvironment (Figure 4C). OCT4 staining showed a progressive increase, becoming clearly positive at day 7 and further enhanced by day 14, indicating acquisition of a stemness phenotype that requires sustained culture in 3D. ROS staining was minimal at early time points but markedly increased by day 14, consistent with metabolic stress accompanying adaptation. Senescence-associated β-galactosidase staining was detectable as early as day 3 and intensified by days 7 and 14, demonstrating that dimensional priming rapidly triggers stress responses that accumulate with time. Finally, we examined how these changes influenced ADSC cytokine secretion. In monoculture, 3D-primed ADSCs displayed lower basal levels of IL-4, IL-6, IL-10, IL-1RA, and TNF-α compared with 2D cultures (Figure 4D). However, in co-culture with CFPAC-1 cells, 3D-primed ADSCs exhibited a distinct secretory response characterized by increased IL-6 and TNF-α and reduced IL-10 (Figure 4E). These findings suggest that while 3D priming suppresses basal cytokine release, it enhances pro-inflammatory responsiveness when ADSCs interact with tumor cells. Together, these results demonstrate that dimensional priming reprograms ADSCs by downregulating CAV-1, reshaping gene expression, and inducing time- and stiffness-dependent adaptation, which in turn alters their cytokine response during tumor co-culture.

### 3.5. 3D-Primed ADSCs Promote PDAC Tumor Growth and Invasiveness In Vivo

**Figure 5:**
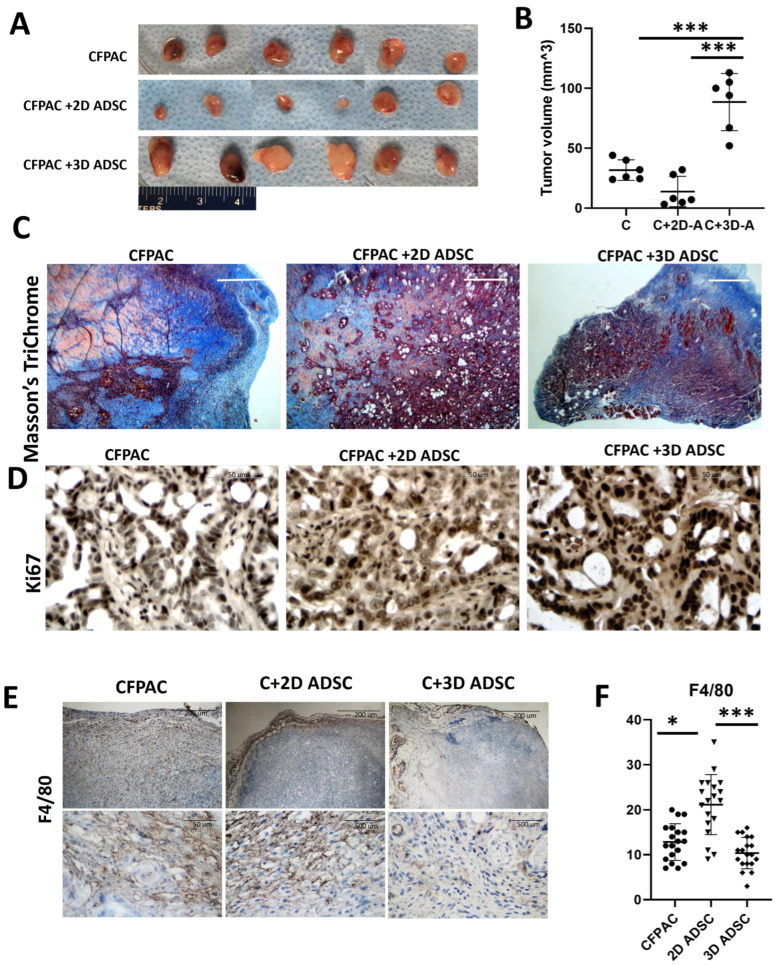
Dimensional priming of ADSCs differentially affects PDAC tumor growth, histology, and immune infiltration in vivo. (**A**) Representative gross images of tumors harvested 28 days after subcutaneous implantation of CFPAC-1 cells alone, or co-injection with ADSCs primed under 2D or 3D conditions. Scale bar = 5 mm. (**B**) Tumor weights at the time of harvest were measured using an analytical balance. Bars represent mean ± SEM from *n* = 6 mice per group. (**C**) Masson’s trichrome staining of paraffin-embedded tumor sections to assess Col-Tgel carrier integrity and invasion into surrounding tissue. Scale bar = 200 μm. (**D**) Immunohistochemical staining for Ki-67 to evaluate tumor cell proliferation. Sections were incubated with anti-Ki-67 antibody, HRP-conjugated secondary detection, and hematoxylin counterstain. Scale bar = 50 μm. (**E**) Immunohistochemical staining for the pan-macrophage marker F4/80 to assess tumor-associated macrophage infiltration. Scale bar = 50 μm, 200 μm, 500 μm. (**F**) Quantification of F4/80-positive cells was performed by counting positive cells in five randomly selected high-power fields per tumor section. Data represent mean ± SEM. * *p* < 0.05, *** *p* < 0.001.

To evaluate the role of dimensional priming on PDAC progression in vivo, CFPAC-1 cells were implanted alone or co-injected with ADSCs primed under 2D or 3D conditions (Figure 5A). Tumors formed in all groups but displayed distinct growth patterns. PDAC cells alone generated medium-sized tumors, while co-implantation with 2D-primed ADSCs produced the smallest tumors, indicating a growth-suppressive effect. In contrast, tumors arising from co-injection with 3D-primed ADSCs were consistently the largest, with significantly greater weight compared to both PDAC-alone and 2D-primed groups (*p* < 0.05) (Figure 5B).

Histological analysis using Masson’s trichrome staining further highlighted differences in tumor–matrix interactions (Figure 5C). In the PDAC-alone group, tumors remained encapsulated within the Col-Tgel delivery vehicle, with intact margins and limited invasion. Tumors co-implanted with 2D-primed ADSCs also retained Col-Tgel boundaries, though vacuolated regions and partial matrix remodeling were evident. In striking contrast, tumors from the 3D-primed ADSC group exhibited loss of the Col-Tgel staining. With the loss of encapsulation, the direct invasion into adjacent muscle tissue was observed. Immunohistochemical analysis confirmed enhanced proliferative activity in tumors co-implanted with 3D-primed ADSCs, as reflected by increased Ki-67 positivity compared to both PDAC-alone and 2D-primed groups (Figure 5D). Examination of immune infiltration revealed group-specific differences. Staining for the pan-macrophage marker F4/80 showed the highest macrophage density in tumors co-implanted with 2D-primed ADSCs, intermediate levels in PDAC-alone tumors, and the lowest levels in tumors arising from 3D-primed ADSC co-implantation (Figure 5E,F). These findings suggest that 2D-primed ADSCs promote macrophage recruitment, whereas 3D-primed ADSCs foster a tumor-permissive environment characterized by reduced macrophage infiltration, enhanced proliferation, and aggressive invasion.

## 4. Discussion

Our study demonstrates that the dimensional priming of adipose-derived stromal cells (ADSCs) fundamentally alters their behavior and, in turn, their impact on pancreatic cancer progression. By comparing ADSCs expanded in conventional 2D conditions with those primed in a 3D hydrogel matrix, we found that 3D priming reprograms ADSCs at multiple levels: (i) suppression of basal cytokine secretion but enhanced pro-inflammatory responses during tumor co-culture, (ii) reduced CAV-1 expression and broad transcriptional changes favoring CAF-like and stemness-associated programs, and (iii) functional consequences for PDAC cells, including increased migration in vitro and accelerated growth and invasion in vivo. Importantly, xenograft tumors containing 3D-primed ADSCs displayed increased proliferation, loss of Col-Tgel encapsulation associated with aggressive invasion, and reduced macrophage infiltration compared to tumors with 2D-primed ADSCs, which constrained tumor growth and recruited more macrophages. Together, these findings support a model in which dimensional priming generates a plastic, stress-adapted stromal state that reshapes the tumor microenvironment to favor PDAC progression.

Importantly, the extensive loss of Col-Tgel containment observed in tumors containing 3D-primed ADSCs does not necessarily imply direct, cell-autonomous matrix degradation by these stromal cells. While 2D-primed ADSCs exhibited higher MMP2 activity in vitro, 3D-primed ADSCs promoted aggressive tumor invasion in vivo in the absence of elevated stromal MMP expression. This apparent divergence suggests that matrix breakdown in the 3D-primed condition may arise from emergent tumor–stroma crosstalk, potentially involving tumor cell-derived proteases, recruitment or activation of host inflammatory cells, or localized inflammatory signaling that amplifies matrix degradation. Such non-linear, context-dependent effects are increasingly recognized in vivo, where stromal priming can reshape the tumor microenvironment in ways not predicted by isolated in vitro assays.

Previous studies have highlighted the heterogeneity of CAF populations in PDAC, including subsets with tumor-restraining versus tumor-promoting functions [21]. Our results add dimensional priming as an underappreciated variable that can bias ADSCs toward different stromal fates. In 2D culture, ADSCs retained features that constrained tumor growth, consistent with higher CAV-1 expression [22] and greater macrophage recruitment in vivo. In contrast, 3D priming induced a CAF-like program with markers such as SOX9 and biglycan, associated with ECM remodeling, stemness, and immune modulation [23,24]. This aligns with studies showing that matrix stiffness and 3D architecture promote fibroblast activation and stem-like transitions [11,12].

In vivo, 2D-primed ADSCs recruited more macrophages, while 3D-primed ADSCs reduced F4/80^+^ cell infiltration despite promoting greater tumor growth. This observation reflects the complexity of stromal–immune crosstalk, in which fibroblast subsets can either facilitate immune exclusion or enhance myeloid recruitment [25,26]. In PDAC, macrophages are not strictly polarized but exist along a spectrum shaped by stromal and tumor cues [26]. Future studies using polarization markers and single-cell profiling will be needed to define whether dimensional priming alters macrophage function in addition to recruitment.

Several limitations warrant consideration. First, our in vivo studies were performed in athymic nude mice, which lack a fully functional adaptive immune system. While this model facilitated consistent xenograft formation, the absence of T cells restricts the physiological relevance of our findings. In immune-competent hosts, stromal–immune interactions may play a more dominant role, and it is possible that the tumor-promoting effects of 3D-primed ADSCs could be attenuated—or even reversed—when adaptive immunity is intact. Future studies using syngeneic or humanized mouse models will be essential to capture the complexity of stromal–immune crosstalk [27,28]. Second, we tested only one PDAC cell line (CFPAC-1). Given the marked heterogeneity of PDAC, stromal–tumor interactions are likely to differ across genetic and phenotypic backgrounds. Future experiments incorporating multiple PDAC cell lines, patient-derived organoids, and matched CAFs or ADSCs from diverse donors will be needed to determine whether dimensional priming consistently drives tumor-promoting behavior [29,30]. Third, our immune characterization was restricted to F4/80 staining. While this provided evidence of differential macrophage recruitment, more detailed analyses of macrophage subsets and other immune populations will be required to fully define the immune consequences of dimensional priming [31].

Dimensional priming likely represents only one of many contextual factors that shape stromal behavior in PDAC. Stromal cells continuously integrate signals from hypoxia, inflammatory cytokines, metabolic stress, and direct cancer–stromal contact [32]. Tumor progression itself is dynamic and co-evolutionary, with cancer and stromal cells adapting in parallel [27]. Understanding how dimensionality intersects with these other cues will be critical for designing strategies to therapeutically target the PDAC stroma. Importantly, modulation of matrix context, through biomaterials, pharmacologic targeting of mechanotransduction, or remodeling of stromal signaling, represents a potential therapeutic approach to reprogram tumor-permissive stroma into tumor-restraining states [33,34].

## 5. Conclusions

In summary, our findings reveal dimensional priming as a key determinant of ADSC plasticity and function in PDAC. By altering cytokine secretion, gene expression, and immune modulatory capacity, 3D priming converts ADSCs into a tumor-promoting stromal state that accelerates tumor growth and invasion. These results emphasize the importance of considering dimensional context in stromal biology studies and suggest that targeting priming-dependent pathways could open new therapeutic avenues for modulating the PDAC microenvironment. Overall, our findings identify dimensional priming as a critical regulator of stromal cell plasticity in PDAC and suggest that mechanical reprogramming of the stroma may represent a novel therapeutic target. Future studies should explore how dimensional priming pathways can be therapeutically manipulated to reprogram PDAC stroma toward tumor-restraining phenotypes.

## Data Availability

The data supporting the findings of this study are available from the corresponding author upon reasonable request. All raw data, processed datasets, and analysis scripts can be provided to qualified researchers.
